# CRISPR/Cas9 Technology as an Emerging Tool for Targeting Amyotrophic Lateral Sclerosis (ALS)

**DOI:** 10.3390/ijms19030906

**Published:** 2018-03-19

**Authors:** Ewa Kruminis-Kaszkiel, Judyta Juranek, Wojciech Maksymowicz, Joanna Wojtkiewicz

**Affiliations:** 1 Department of Pathophysiology, School of Medicine, Collegium Medicum, University of Warmia and Mazury, 10-900 Olsztyn, Poland; judyta.juranek@uwm.edu.pl; 2 Department of Neurology and Neurosurgery, School of Medicine, Collegium Medicum, University of Warmia and Mazury, 10-900 Olsztyn, Poland; maksymowicz@interia.pl; 3 Laboratory for Regenerative Medicine, School of Medicine, Collegium Medicum, University of Warmia and Mazury, 10-900 Olsztyn, Poland

**Keywords:** CRISPR/Cas9, ALS, ALS models, ALS therapy

## Abstract

The clustered regularly interspaced short palindromic repeats (CRISPR)/CRISPR-associated protein-9 nuclease (Cas9) is a genome editing tool that has recently caught enormous attention due to its novelty, feasibility, and affordability. This system naturally functions as a defense mechanism in bacteria and has been repurposed as an RNA-guided DNA editing tool. Unlike zinc-finger nucleases (ZFNs) and transcription activator-like effector nucleases (TALENs), CRISPR/Cas9 takes advantage of an RNA-guided DNA endonuclease enzyme, Cas9, which is able to generate double-strand breaks (DSBs) at specific genomic locations. It triggers cellular endogenous DNA repair pathways, contributing to the generation of desired modifications in the genome. The ability of the system to precisely disrupt DNA sequences has opened up new avenues in our understanding of amyotrophic lateral sclerosis (ALS) pathogenesis and the development of new therapeutic approaches. In this review, we discuss the current knowledge of the principles and limitations of the CRISPR/Cas9 system, as well as strategies to improve these limitations. Furthermore, we summarize novel approaches of engaging the CRISPR/Cas9 system in establishing an adequate model of neurodegenerative disease and in the treatment of SOD1-linked forms of ALS. We also highlight possible applications of this system in the therapy of ALS, both the inherited type as well as ALS of sporadic origin.

## 1. Introduction

The clustered regularly interspaced short palindromic repeats (CRISPR)/CRISPR-associated protein-9 nuclease (Cas9) is a novel genome editing tool that has recently revolutionized the field of human genetic engineering. This system naturally serves as an adaptive defense mechanism in bacteria and archaea against foreign nucleic acids such as plasmids and bacteriophages [1]. The CRISPR/Cas9 system belongs to a programmable nuclease-based genome editing technology together with zinc-finger nucleases (ZFNs) and transcription activator-like effector nucleases (TALENs) [2,3,4]. The programmable nucleases are able to generate mutations at a specific genomic location. These endonucleases serve as genomic scissors, creating double-strand breaks (DSBs) and, therefore, assure precise genome editing [5]. The disruption in the DNA sequence triggers various repair mechanisms, such as non-homologous end joining (NHEJ) and homology-directed repair (HDR), leading to the induction of specific knock-outs or knock-ins [6]. Scientists can exploit the cell’s endogenous DNA repair pathways to generate mutations at the desired genome sites. However, ZFNs and TALENs operate through protein–DNA interactions and targeting new genomic loci requires engineering and cloning a new protein, which makes both of these methods laborious and difficult [7]. In contrast, CRISPR/Cas9 is categorized as a type II system of CRISPR/Cas, utilizing guide RNA (gRNA) to target specific DNA sequences and engages Cas9 as a nuclease [8]. The specificity of both ZFNs and TALENs can be improved by increasing the number of zinc fingers or TALE proteins while the specificity of CRISPR/Cas9 is determined by a 20 bp guide RNA. Furthermore, since the targeting specificity of ZFNs and TALENs is dependent only on protein–DNA interaction, the off-target effects cannot be fully predicted based on DNA sequence homology. In contrast, the off targets of CRISPR/Cas9 can be more earnestly predicted based on sequence homology due to the fact that CRISPR/Cas9 recognizes targets according to Watson–Crick base-pairing rules. To sum up, compared to traditional programmable nuclease-based genome editing technologies, the CRISPR/Cas9 system has numerous benefits such as ease in target design, the facility to target multiple sites in one step, and predictable off-target sites [9]. Thus, since CRISPR/Cas9 is based on an RNA-guided mechanism, it is better for high-throughput applications and is being applied in various research fields such as biotechnology and medicine. As the pace of CRISPR/Cas9 research has accelerated, its potential has also been explored in both disease modeling and gene therapy tailored treatment of incurable diseases with pathomechanisms that have not yet been fully uncovered, including amyotrophic lateral sclerosis (ALS). ALS is characterized as having a relatively slow progression towards being fully explained in terms of the factors and mechanisms that are implicated in the neurodegeneration [10]. Moreover, there is still a lack of an effective treatment that would significantly prolong the lifespan of ALS-affected people. These arguments make it important to develop systemic models that faithfully mimic this disorder in order to help in the development of effective therapeutics.

## 2. CRISPR/Cas9 System

The discovery of the CRISPR loci began in 1987 when Ishino et al. [11] first reported the presence of mysterious DNA sequences in the genome of *Escherichia coli*. This stretch of DNA consists of short direct repeats separated by short unique sequences. It was found later that CRISPR/Cas machinery functions analogously in bacteria and archaea to the adaptive immune system in vertebrates, providing a genetic library (memory) of phages and plasmids that previously have invaded the bacteria. Resistance against foreign nucleic acids after the subsequent infection is assured due to the enzymatic activity of the Cas proteins [1].

**Figure 1:**
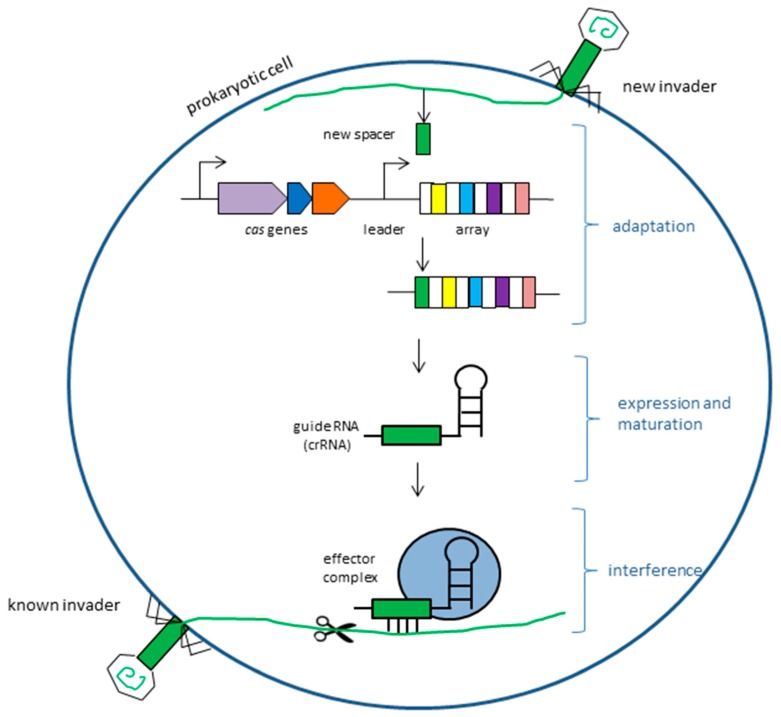
The three steps of CRISPR/Cas (clustered regularly interspaced short palindromic repeats/CRISPR-associated protein-9 nuclease) immunity.

CRISPR/Cas-mediated immunity acts in three steps: adaptation, expression with maturation, and interference. In the first adaptive phase, bacteria or archaea incorporate short foreign sequences (protospacers) into the host genome generating repeat-spacer motifs. In the next phase, transcription of the repeat-spacer sequences yields precursor CRISPR RNAs (pre-crRNAs) that after maturation become crRNA molecules, which are able to recognize and target invading genetic material in the interference step. Due to the activity of Cas proteins, the invading nucleic acid can be degraded [12,13] (Figure 1).

Among the three existing types of CRISPR/Cas systems, the type II system involving Cas9 proteins has proven to be the most promising genome engineering tool [8,14]. The type II CRISPR/Cas9 originates from *Streptococcus thermophilus* and is able to specifically cleave double-stranded DNA [15]. The site-specific DNA disruption in this system requires both base-paired complementarity of CRISPR RNA (crRNA) to the target sequence and a short DNA sequence called a protospacer-adjacent motif (PAM). Cas9 derived from various species recognizes different PAMs, and, therefore, Cas9 originating from *Streptococcus pyogenes* requires 5′-NGG-3′ sequences adjacent to the target locus [16]. Jinek et al. [14] revealed that Cas9 from *Streptococcus pyogenes* can be directed by two RNAs: CRISPR RNA (crRNA) and trans-activating RNA (tracrRNA) constituting crucial components of the type II CRISPR/Cas system. Moreover, dual-tracrRNA:crRNA can be successfully replaced by an engineered single guide RNA (sgRNA) to simplify the task of programming Cas9 [14].

**Figure 2:**
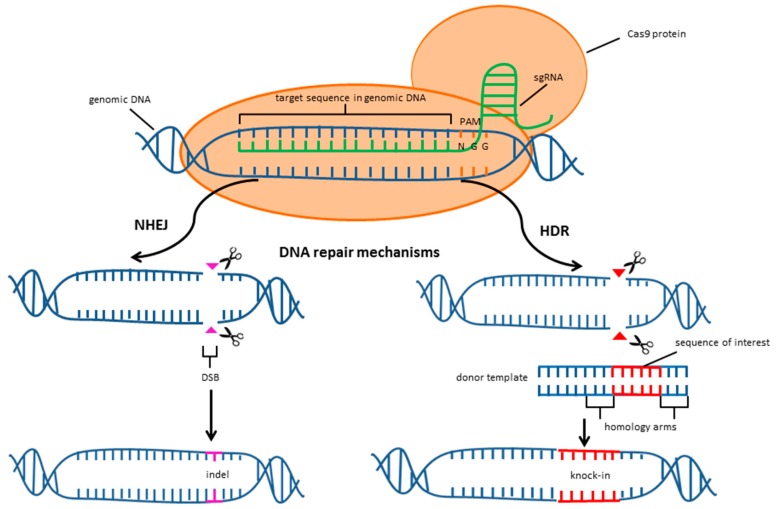
CRISPR/Cas9 mediated cleavage of genomic DNA and two major repair pathways.

The double-strand breaks (DSBs) induced by the Cas9-sgRNA complex are repaired by either of two major distinct pathways: nonhomologous end joining (NHEJ) or homology-directed repair (HDR). Both of them are able to operate in almost every cell type and organism [17] (Figure 2). NHEJ is the prevailing pathway that can be performed in all phases of the cell cycle. Due to the flexibility of DNA ligase IV mediating NHEJ, there is a noticeable heterogeneity of processed ends leading to insertions or deletions (indels) around cleavage sites. If such mutations are introduced into the coding exon, it can result in a frameshift and premature codon stoppage, leading to gene knock-out. The NHEJ process is error-prone and can cause unforeseeable mutations [18]. Although the alternative pathway, HDR, occurs less frequently and is mainly restricted to specific phases of the cell cycle, it enables precise DNA repair [19,20]. HDR requires exogenous and homologous DNA to replace or insert DNA sequences into target loci [21]. Thus, HDR is a desirable tool for generating specific gene corrections or insertions of fluorescent markers or protein tags enabling the confirmation of introduced mutations [22]. Various approaches have been taken to enhance HDR in order to improve the rate of high-fidelity genome edits. Both of the above-mentioned DNA repair pathways compete with each other and, therefore, the inhibition of NHEJ can increase the rate of HDR. Maruyama et al. [23] enhanced HDR through antagonizing the crucial enzyme involved in NHEJ–DNA ligase IV. Furthermore, the nuclease platform has an influence on HDR/NHEJ ratios and the inactivation of the Cas9 nuclease domains elevates the ratio of HDR [21,24].

## 3. Limitations of the CRISPR/Cas9 System

The simplicity of the CRISPR/Cas9 system encourages the exploration of its potential to generate animal models or to treat severe and incurable human diseases. However, potential users need to consider several issues that limit the feasibility of this system.

The guide RNA of the system does not need to fully match the target sequence for its recognition and cleavage [14]. This characteristic feature of the system indicates the existence of off-target effects, which constitute its major hurdle. Off-target events include off-target recognition and digestion as well as other uncontrollable consequences that may lead to the generation of unpredictable mutations. Thus, the off-target activity of Cas9 depends on the uniqueness of the target sequence since Cas9 is able to tolerate a few nucleotide mismatches [25]. Numerous studies have been conducted to evaluate the potential off-target effects. It was revealed that the mismatches at the 5′-end are better tolerated since the sequence at the 3′-end is significant for target identification [26]. Moreover, the frequency of off-target cleavage can be similar to on-target mutation and, therefore, the application of the system for therapeutic purposes might be questionable [25]. Hence, various approaches should be undertaken to verify possible off-target events in order to reduce undesirable mutations.

The other concern is the delivery of sgRNA and Cas9. Different strategies involve viral and non-viral vectors to introduce components of the system. Lentiviral (LV) or adenoviral vectors (AV) are common and effective tools used as delivery systems for sequence-specific sgRNA and Cas9 nucleases [27,28,29]. Nevertheless, viral vectors have several drawbacks such as limited cargo capacity and tissue tropism confined to specific organs. For example, adeno-associated vectors (AAVs) possess a tropism to muscle, liver, brain, and eye [30]. Certainly, recent advances in genome editing tools have focused on overcoming this issue. Furthermore, novel approaches such as the generation of off-target effects have been developed to circumvent the main disadvantage of the entire system. Consequently, pre-packaging of the Cas9 protein simultaneously with sgRNA into a lentivirus can diminish these side effects [31]. Viral vectors used for therapeutic applications are rearranged to impede replication and avoid virulence. Nevertheless, even after rearrangement, undesirable harmful consequences may occur [31]. Non-viral approaches can be used as a safer alternative to deliver the tools for gene editing. These approaches include electroporation, nucleofection or microinjection using glass microcapillaries, and Lipofectamine [32,33,34]. Li et al. [35] have recently discovered an artificial virus that enables the effective loading of the CRISPR/Cas9 system in mice. Additionally, this newly found delivery strategy is even more reliable than lipofectamine, assuring precise gene cleavage.

Although the immunogenicity of viral vectors used for in vivo delivery has been taken into consideration, little is known about the ability of the bacterial Cas9 protein to trigger an immune response in humans [36]. Certainly, the potential adverse effects of Cas9 derived from bacteria need to be fully elucidated and circumvented in order to develop therapeutics based on CRISPR/Cas9. For example, humanizing the intrinsic peptide fragments is a potential strategy to reduce the immunogenicity of Cas9 [5].

## 4. Improving the Specificity and Efficiency of the CRISPR/Cas9 System

The above-mentioned hurdles of the CRISPR/Cas9 system reveal its imperfection. Nevertheless, much effort has already been made to increase the specificity and efficiency of the system. Although the risk of off-target mutagenesis dramatically undermines the CRISPR/Cas9 system, various approaches have been undertaken to avoid undesirable mutations and increase on-target efficiency. The potential off-target effects can be diminished from the beginning of the experiment through the selection of an appropriate target site and the designing of the sgRNA. It has been revealed that the GC (guanine–cytosine) content of sgRNA has an influence on the rate of off-targets [37].

One of the approaches to elicit a higher specificity of this technique can be a simultaneous expression of two guide RNAs targeting opposite DNA strands [23]. Paired gRNAs reduce off-target mutagenesis whereas inactivation of one of the Cas9 nuclease domains (Cas9n, nickase) results in high-efficiency deletions [38,39]. The application of a single Cas9 nickase does not need to be restricted to deletions only. Gao et al. [40] have recently employed a single Cas9 nickase (Cas9n) to insert genes at a specific target site in cattle genomes.

Additionally, the off-target cleavage can be significantly diminished without loss of on-target efficiency by using truncated guide RNAs (tru-gRNAs). The truncated guide RNA fragments recognize shorter target sequences of less than 20 nucleotides [41]. This approach poses a feasible and effective approach to increase the specificity of both Cas9 nuclease and paired nickase.

Another strategy to improve the on-target mutagenesis with less off-target effects is the fusion of catalytically inactive Cas9 to *Fok*I nuclease (fCas9). Two monomers of fCas9 are able to modify target DNA with at least a 4-fold higher specificity than paired Cas9 nickases (Cas9n), retaining the efficiency comparable to that of paired Cas9n [42]. Furthermore, for further improvement of genome editing specificity, Wyvekens et al. [43] concatenated two distinct strategies such as the generation of *f*Cas9 and the truncation of gRNAs. Such a combination provides highly efficient and specific genome modification.

The application of the CRISPR/Cas9 system for therapeutic purposes requires a safe and efficient method of provisioning the components. Delivering the *Cas9* gene and sgRNA in plasmids carries the risk of a prolonged expression of RGENs, which increases the number of off-target events. Thus, in vivo therapy requires a “hit and run” strategy that depends on the introduction of Cas9 protein instead of the *Cas9* gene. This solution assures immediate induction of mutations such as insertions or deletions at target sites and rapid degradation of RGEN ribonucleoproteins leading to off-target reduction [32].

## 5. The Application of CRISPR/Cas9 for Modeling Neurodegenerative Disease

The establishment of a good animal model for neurodegenerative diseases that faithfully simulates human illness requires mimicry of the symptoms as well as developing pertinent neuropathological features. A successfully-generated animal model provides a significant contribution to the understanding of disease pathophysiology and helps to develop therapeutic and preventative strategies. Genetic conditioning of neurodegenerative diseases makes it possible for them to be imitated in animal models by using genome engineering tools. Even though the generation of genetically modified animal models has been abundant in the last two decades, most of them referred to small animals such as invertebrates or rodents [44,45,46]. Certainly, the superiority of large animal models over small ones is undisputed since their anatomy and physiology are more similar to humans and, therefore, can more accurately reproduce the clinical manifestations of a specific disease.

The development of the CRISPR/Cas9 tool has opened the door for the advancement of new model systems in neuroscience. These models include in vivo animal models as well as stem cell-derived models for studying “a disease in a dish”. The traditional strategies are based on microinjection of genetically modified embryonic stem cells into blastocysts for germline transmission. Unfortunately, such approaches require much time and effort and might be inefficient in introducing genome modification. Additionally, the traditional methods are not applicable for generating large animal models. The CRISPR/Cas9 system has many advantages in modeling neurodegenerative diseases and, therefore, might help to avoid the above-mentioned problems of traditional technologies [47].

The application of the CRISPR/Cas9 system as a novel tool for modeling neurodegenerative diseases has been facilitated due to its ability to generate mutations in endogenous genes. On the other hand, traditional methods lead to the expression of mutant genes under exogenous promoters and, therefore, are not useful for modeling the diseases. Furthermore, the CRISPR/Cas9 system is able to introduce mutations in one or two alleles in order to mimic heterozygous or homozygous knock-outs of a given gene. Targeting both alleles results in a null mutation in the model organism. This is significantly beneficial for generating large animal models since the generation of homozygous mutants by mating two heterozygous mutants is highly time consuming. Additionally, the disruption of both alleles in female animals enables the complete loss of function of a given gene when the disease is linked to the X chromosome [47].

The CRISPR/Cas9 system has been recently used for engineering the pig genome in order to generate a human Parkinson’s disease model. In this study, three different genomic loci were targeted including *parkin*, *DJ-1*, and *PINK1* genes by multiple sgRNAs co-injected into pronuclear embryos of Bama miniature pigs. The genetically modified piglets remained healthy with a normal growth rate. Although the 10-month-old mutant pigs did not display typical symptoms of Parkinson’s disease, the influence of the triple-gene targeted modification was confirmed on a molecular level. Immunofluorescence and Western blot analysis revealed the absence of DJ-1 protein expression in the fibroblasts, confirming the inactivation of the DJ-1 gene on both alleles. The expression of the *parkin* and the *PINK1* gene determined by real-time RT-PCR was also significantly decreased in the fibroblasts of genetically modified pigs when compared with wild-type cells. Moreover, despite the high number of sgRNAs that were employed, the trio-based whole-genome sequencing analysis demonstrated a low incidence of off-target effects [48]. The results of this study have revealed the enormous potential of the CRISPR/Cas9 system in the modification of multiple genes in pigs and the results have confirmed the utility of the system in modeling neurodegenerative diseases.

The CRISPR/Cas9 system was also successfully employed to target monkey genomes. The co-injection of Cas9 mRNA and sgRNAs into one-cell-stage embryos enabled concurrent modification of two target genes (Ppar-γ and Rag1) in one step. Fortunately, the off-target mutagenesis was not identified by a comprehensive analysis. Taken together, the study confirmed the reliability of the system for simultaneous disruption of two genes that led to the generation of a genetically modified cynomolgus monkey [49].

In the context of ALS, CRISPR/Cas9 seems to be an attractive tool since ALS is a rapidly progressive, fatal neurodegenerative disease that can be the result of a combination of genetic and environmental factors [50]. Inherited cases of ALS constitute about 10% of all cases. Of those, 20% are caused by a mutation in the Cu/Zn superoxide dismutase (*SOD1*) gene. The remaining familial ALS cases are associated with mutations in various genes among which the most common are mutations in the 43-kDa TAR DNA-binding protein (*TARDBP*) gene [51] or in the fused/translocated liposarcoma (*FUS*) gene [52]. C9orf72-related ALS is also noteworthy as it is age dependent and is inherited in an autosomal dominant manner. Patients with ALS or frontotemporal lobar degeneration (FTLD) exhibit pathogenic C9orf72 G4C2 hexanucleotide repeat expansion [53].

Taking into consideration the genetic background of familial ALS cases, several studies have been conducted to develop transgenic ALS models, among which the recently discovered CRISPR/Cas9 system holds great promise for a simple, feasible, and inexpensive method for generating model systems. Armstrong et al. [54] have recently introduced point mutations into the zebrafish *TARDBP* and *FUS* genes using the CRISPR/Cas9 system to develop an ALS model in zebrafish. Although CRISPR/Cas9-mediated NHEJ led to the generation of INDELs, it failed to create single nucleotide substitutions. Nevertheless, co-injection of single-stranded oligodeoxynucleotide donor templates (ssODN) containing point mutations with gRNA and Cas9 mRNA resulted in HDR integration of the two missense mutations (*TARDBP*^A379T^, *FUS*^R536H^). Thus, this approach has proven that homology-directed repair (HDR) following CRISPR/Cas9 enables the generation of knock-in lines that imitate disease-causing point mutations in humans using zebrafish *TARDBP*^A379T^ (*TARDBP*^A382T^) and *FUS*^R536H^ (*FUS*^R521H^). Since CRISPR/Cas9-mediated NHEJ has limited potential to model phenotypes, HDR is recommended for that purpose. Unfortunately, CRISPR/Cas9-mediated HDR can be problematic due to its low efficiency and therefore various novel approaches have been developed to increase efficiency, likely leading to a better integration of the donor template. It might seem that the expression of a human gene with diminished homology to the ortholog in a phylogenetically distant species can be insufficient and too biased to reflect the human disease. Fortunately, the CRISPR/Cas9-mediated breaks and HDR ssODN template knock-in successfully avoids this restriction by editing the endogenous gene. Secondly, this novel technique saves time and labor since genomic integration in zebrafish can be evaluated within two days [54].

Additionally, CRISPR technology has been used to develop pathogenic variants of ALS in mice. By taking advantage of the system’s ability to create several patient-specific mutations in genes coding different domains of a single protein, it is now possible to recapitulate the disease caused by various mutations [55]. The CRISPR/Cas9 system has been successfully used to establish C9orf72-deficient mice [56] since hexanucleotide repeat expansion in the *C9orf72* gene has been recently discovered as a major cause of frontotemporal lobar degeneration (FTLD) with amyotrophic lateral sclerosis (ALS) [57]. Furthermore, this genome engineering tool is able to generate knock-downs of particular genes in the already well-known *hSOD1*^G93A^ ALS mouse model. The genes encoding several neurotrophic factors are being investigated in the context of ALS due to their therapeutic potential in neurodegenerative diseases. The gene encoding insulin-like growth factor 1 (IGF1) was knocked-down in this mouse model in order to observe and verify the processes that are influenced by IGF-1 [58].

This versatile genome editing tool has recently received attention for its potential in cellular models of ALS. The applicability of the CRISPR/Cas9 system for studying a “disease in a dish” that employs the patient’s own derived induced pluripotent stem cells (iPSCs) was highlighted. Mutihac et al. [59] from the University of Oxford applied the CRISPR/Cas9 system to confirm models of amyotrophic lateral sclerosis (ALS) and frontotemporal dementia (FTD). This research involved the generation of induced pluripotent stem cell lines derived from ALS patients with the C9orf72 repeat expansion. One line was repaired by excision of the mutation using CRISPR/Cas9 to unravel crucial pathological processes in ALS. The other line of iPSC-derived motor neurons was investigated to determine pathological features that included disturbed Ca^2+^ homeostasis and decreased cell survival corresponding to a diminished rate of anti-apoptotic protein Bcl-2. Overall, it was delineated that iPSC-derived motor neurons are valuable cellular models of neurodegenerative disorders and are a novel genome engineering tool that can facilitate discoveries.

## 6. The Application of the CRISPR/Cas9 System for Amyotrophic Lateral Sclerosis Therapy

The fatal outcome of ALS accelerates the urgent need for the discovery of novel therapeutic approaches that may lead to the development of a cure for this devastating disease. Conventional therapies are focused on symptoms and are not able to eliminate the pathology underlying the clinical manifestation. The potential of gene therapy to permanently affect the intracellular processes is promising for the treatment of incurable neurodegenerative diseases. Genome engineering tools are used to target other inherited movement disorders such as Parkinson’s disease both in preclinical studies [60] and in clinical trials [61]. Although most cases of ALS are of a sporadic origin with multiple etiologic factors, there are currently over 20 genes underlying the ALS neuropathology, among which more than seven have been discovered recently. Hence, the question arises of whether modifications of a causative gene can result in impaired progression of the disease in humans. The studies conducted on a SOD1^G93A^ mouse model have revealed that silencing of the mutant *SOD1* gene using both interfering RNA (iRNA) [62] and artificial microRNA (miR-SOD1) [63] delayed the onset of ALS and prolonged the lifespan of these mice. Both of these studies have used viral vectors as vehicles to carry gene silencing “devices.” Similar to iRNA and microRNA, CRISPR-mediated genome editing has recently revealed its enormous potential to treat ALS. Gaj et al. [64] demonstrated that the CRISPR/Cas9 can be applied to modify mutant expression in the G93A-SOD1 mouse model of ALS following in vivo delivery using an adeno-associated virus vector (AAV). Gene disruption contributed to the reduced expression of mutant SOD1 protein in the lumbar and thoracic spinal cord leading to enhanced motor function and diminished muscle atrophy. Importantly, ALS mice treated by CRISPR/Cas9 had increased survivability of motor neurons, delayed disease onset, and a prolonged lifespan compared to controls. This study has significantly confirmed the potential of CRISPR/Cas9 as a successful tool for the treatment of SOD1-linked forms of ALS and other neurodegenerative diseases.

The application of the CRISPR/Cas9 system in the therapy of ALS currently has a dual purpose: gene therapy for inherited ALS and the expression of genes encoding neurotrophic factors (NTFs). Recently, the University of Massachusetts Medical School received a grant to develop two gene therapies to target the mutant *C9orf72* gene, the common cause of familial ALS. One of these approaches involves the *CRISPR*/*Cas9* gene editing tool to remove the mutant gene through enzymatic cleavage; the other strategy is focused on delivering RNA in order to reduce C9orf72 expression [65].

The second approach of using CRIPSR/Cas9 technology in ALS therapy is based on the meaningful potential of neurotrophic factors to exert neuroprotective effects on damaged motor neurons in both familial and sporadic ALS cases. Although NTFs emerged as promising candidates for ALS treatment in the early 1990s, initial treatments proved to be ineffective especially due to delivery issues [66]. More controlled and targeted delivery of neurotrophins is required to avoid side effects including unknown interactions of these proteins. The innovative NTF delivery tools such as neurotrophic factor protein supply through an engineered viral vector or the transplantation of cells modified to express NTFs might help to circumvent some obstacles [67]. Nevertheless, the safety and efficacy of using viral vectors should be fully elucidated before clinical translation. The genetically engineered stem cells represent a promising vehicle to deliver NTFs. Using genetically modified mesenchymal stem cells (MSCs) to express neurotrophic factors might be a less invasive method of administration and may enhance the neuroprotective effects of MSCs on impaired motor neurons. Thus, engrafting such cells can be dually beneficial. Preliminary findings from The Netherlands, obtained by van den Akker and coworkers [68] highlight the potential of the CRISPR/Cas9 system in modifying MSCs. They revealed that MSCs are able to steadily express CRISPR/Cas9 components along with the standard differentiation features of these cells. However, the studies should be extended to confirm the possibility for a knock-in of the desired gene into these stem cells. Once the expression of a gene is evaluated and optimized, the cells can be engrafted into the central nervous system regions to act locally in providing neurotrophic factors. Since the combination of several growth factors might be more successful in the context of ALS treatment, stem cells equipped with multiple *NTFs* genes would be another possibility [69].

## 7. Conclusions and Challenges

The CRISPR/Cas9 system makes genome engineering technology feasible for application in many fields including human diseases. Current research on this innovative genome editing tool and strategies to improve its drawbacks has laid the groundwork for future clinical work in neurodegenerative diseases. It has opened new avenues for studying the complexity of neurodegeneration in both in vivo and in vitro model systems. The ability of the system to establish large animal models that recapitulate specific human diseases improves our understanding of complex mechanisms involved in disease pathology. Lessons learned from modeling neurodegenerative diseases in large animals using CRISPR/Cas9 give a confirmation that despite several limitations of the system, this new technology is able to effectively modify animal genomes with the absence of off-target mutagenesis. Targeting multiple genes in the same cell by CRISPR/Cas9 technology enables the study of synergistic effects due to the loss of crucial genes. Although in the context of ALS research the CRISPR/Cas9 system is still rather limited to generating small animal models or improving the in vitro models, results obtained from studies mentioned in this review may facilitate the field of modeling ALS.

Moreover, the feasibility of the CRISPR/Cas9 technology to treat ALS revealed recently in mice gives enormous hope to patients suffering from ALS. Although such approaches are currently restricted to reducing the expression of mutant proteins in inherited cases of ALS in mice, the system might bring breakthroughs in both the familial and the sporadic origin of the disease in the future.

The possibilities of CRISPR-mediated genome editing summarized in this review both in modeling and possible treatment of ALS have begun a new era in ALS research and the CRISPR/Cas9 system has emerged as a feasible tool to achieve what has seemed impossible for decades.
